# Optimizing Sequencing Resources in Genotyped Livestock Populations Using Linear Programming

**DOI:** 10.3389/fgene.2021.740340

**Published:** 2021-10-22

**Authors:** Hao Cheng, Keyu Xu, Jinghui Li, Kuruvilla Joseph Abraham

**Affiliations:** ^1^ Department of Animal Science, University of California, Davis, Davis, CA, United States; ^2^ Department of Economics, FEARP, University of São-Paulo, Ribeirão Preto, Brazil; ^3^ Department of Computer Science-ICMC, University of São Paulo, São Carlos, Brazil

**Keywords:** dairy cattle, sequencing, linear programming, haplotypes, selection

## Abstract

Low-cost genome-wide single-nucleotide polymorphisms (SNPs) are routinely used in animal breeding programs. Compared to SNP arrays, the use of whole-genome sequence data generated by the next-generation sequencing technologies (NGS) has great potential in livestock populations. However, sequencing a large number of animals to exploit the full potential of whole-genome sequence data is not feasible. Thus, novel strategies are required for the allocation of sequencing resources in genotyped livestock populations such that the entire population can be imputed, maximizing the efficiency of whole genome sequencing budgets. We present two applications of linear programming for the efficient allocation of sequencing resources. The first application is to identify the minimum number of animals for sequencing subject to the criterion that each haplotype in the population is contained in at least one of the animals selected for sequencing. The second application is the selection of animals whose haplotypes include the largest possible proportion of common haplotypes present in the population, assuming a limited sequencing budget. Both applications are available in an open source program LPChoose. In both applications, LPChoose has similar or better performance than some other methods suggesting that linear programming methods offer great potential for the efficient allocation of sequencing resources. The utility of these methods can be increased through the development of improved heuristics.

## 1 Introduction

The discovery of genome-wide single-nucleotide polymorphisms (SNPs) and effective ways to assay them has revolutionized genetic analyses of quantitative traits in animal breeding (VanRaden, 2008; Hayes et al., 2009; VanRaden et al., 2009; Habier et al., 2010; Wolc et al., 2012). In conventional breeding programs, low-cost SNP array data are routinely used in genomic selection to estimate breeding values. Compared to SNP arrays, the use of whole-genome sequence data generated by next-generation sequencing technologies (NGS) has great potential in livestock populations for causal mutation detection (Daetwyler et al., 2014) in genome-wide association studies and more stable or accurate prediction of breeding values in genomic selection (Meuwissen and Goddard, 2010).

As sequencing is expensive, there is considerable interest in extracting as much information as possible by sequencing a limited number of animals and then imputing to other animals. Some strategies for allocating limited sequencing resources in genotyped livestock populations require that certain key individuals be sequenced at high coverage (Druet et al., 2014) while others consider sequencing a large number of animals at lower coverage (VanRaden et al., 2015; Li et al., 2011). Sequencing a large number of animals at low coverage offers certain advantages; in VanRaden et al. (2015), the authors comment on the improvement in genotype imputation attained through increasing the number of animals sequenced at low coverage, and in Li et al. (2011), the authors point out the benefits of sequencing many individuals at low coverage in complex trait association studies. The methods we present in this paper are better suited for selecting animals to be sequenced when selecting a large number of animals at low coverage.

The choice of which animals to sequence has been studied from the perspective of maximizing the efficiency of whole genome sequencing experiments. To this end, in Bickhart et al. (2016), the authors discuss the problem of finding a subset of animals of limited size which covers all of the haplotypes present in a given population with a frequency above a predefined threshold. The selection of animals for sequencing based on haplotypes and their frequencies has also been extensively discussed in Butty et al. (2019). The methods we will present for the selection of animals assume that a low-density haplotype library has been constructed, and that low-density haplotype information on all animals is available. We will not make use of pedigree information (Ros-Freixedes et al., 2020), or discuss overall imputation accuracy (Gonen et al., 2017; Ros-Freixedes et al., 2017). We will however assume that in order to recover through imputation a given haplotype in any animal, at least one animal containing that haplotype must be sequenced. This requirement imposes a number of constraints to be simultaneously satisfied.

Selecting the best set of animals in a population capable of satisfying certain constraints is an optimization problem in a space whose dimension is equal to the total number of animals in the population. In practical applications the optimization must be performed for tens of thousands of animals subject to tens of thousands of constraints. Despite the large dimension of the search space, and the large number of constraints, the optimization problem we are trying to solve here can be addressed through the use of a well-established set of techniques known as linear programming [for a review see (Luenberger and Ye, 2015)]. Linear programming has been previously applied in animal and plant breeding to optimize breeding decisions (Diaz et al., 1999; Moeinizade et al., 2019), but not to the allocation of sequencing resources. In this paper, we will study the application of linear programming to the allocation of sequencing resources to address two different questions which may arise in breeding programs.

The first question is to determine the minimum number of animals needed to permit sequence imputation into all other members of the population without loss of haplotype diversity, and then to identify these animals. If a subset of animals which contains all the haplotypes in the population can be identified, then the animals in this subset can be considered to be a starting point for sequencing to eventually permit imputation into the entire population. In order to minimize overall costs, we will attempt to find the smallest subset of animals with this property.

In practice, however, even after the smallest subset of such animals has been identified, it may not be possible to sequence all the animals identified in this manner due to budget constraints. It may then become necessary to choose a limited subset of animals carrying haplotypes which may be considered to be representative of the population on the basis of being more frequent. Identifying this limited subset of animals is the second question which will be addressed in this paper. The objectives of this paper are to study the use of linear programming models to address all of the questions described above, and to compare the performance of linear programming methods with previously published methods.

We will describe in detail the application of our linear programming based method, LPChoose, and also compare the numerical results obtained from LPChoose to several approaches including IWS (Bickhart et al., 2016; Butty et al., 2019), AHAP (Druet et al., 2014; Bickhart et al., 2016) and AlphaSeqOpt (Gonen et al., 2017). This numerical comparison between the results obtained by different methods is completely based on observed haplotype frequencies, we do not make use of ancestral haplotype frequencies or haplotypes frequencies from databases in our selection criteria for animals to sequence. We will also explain how some key criteria of the HSH and GDI schemes in Butty et al. (2019) and the IWS scheme in Bickhart et al. (2016) for the selection of individuals can be understood in the language of linear programming. Two important questions related to imputation, haplotype phasing and resolution, which are addressed in Gonen et al. (2017), and in Ros-Freixedes et al. (2017), will not be discussed here.

## 2 Methods

We now establish some notation which will be used throughout this paper. For each animal we associate an indicator variable *x*_*j*_ such that *x*_*j*_ ∈ (0, 1) and *j* ∈ (1, 2, …, *n*) where *n* is the total number of animals. *x*_*j*_ = 1(0) means that the animal *j* is selected (not selected) for sequencing. We next introduce binary constants *a*_*ij*_ where *i* ∈ (1, 2, …, *p*) and *p* is the total number of unique haplotypes. *a*_*ij*_ = 1(0) if animal *j* carries haplotype *i* (or not). Since we are solving systems of inequalities in which the variables are binary valued, we actually work within the framework of integer linear programming, which is more restricted than linear programming.

The first application of integer linear programming we consider is to identify the minimum number of animals for sequencing while meeting the criteria that each haplotype is contained in at least one of the animals selected for sequencing. If haplotype *i* is carried in at least one animal in the population, then we require that $\sum_{j=1}^{n}a_{ij}x_{j}\geq 1$ . This condition can be satisfied only if *x*_*j*_ = 1 for at least one value of 1 ≤ *j* ≤ *n*. A similar constraint must hold separately for all *i* ∈ 1, 2, …, *p*.

In order to minimize the number of animals to be sequenced, we additionally require $z_{1}=\sum_{j=1}^{n}x_{j}$ , where *z*_1_ is the number of selected animals, to be as small as possible. These inequalities can be collectively written as.

$$\begin{array}{ccc} \text{minimize}\; & z_{1}=\overset{n}{\underset{j=1}{\sum }}x_{j},\;x_{j}\in 0,1 & \end{array}$$ (1)

$$\begin{array}{ccc} \text{subject to the constraints}\; & \overset{n}{\underset{j=1}{\sum }}a_{ij}x_{j}\geq 1,\;i=1,2,3,\cdots \;,p & \end{array}$$ (2)

Equation 1 is the objective function to minimize the total number of selected animals. Equation 2 is the set of constraints which ensures that each haplotype is present in at least one of the animals selected for sequencing.

The objective function and the constraints can also be written in matrix notation as follows.

$$\begin{array}{ccc} \text{minimize}\; & z_{1}=1^{T}x, & \end{array}$$ (3)

$$\begin{array}{ccc} \text{subject to}\; & Ax\geq 1 & \end{array}$$ (4)

where **A** is the matrix of values *a*_*ij*_ and ***x*** denotes the vector of *x*_*i*_ values. This system of inequalities can be solved by integer linear programming. The solution for *x* will contain some values equal to zero and others equal to one. The *x*_*j*_ which are equal to 1 correspond to the animals which should be sequenced.

The second question, to select a fixed number of animals with most common haplotypes, can also be addressed using integer linear programming. In order to prioritize animals with more frequent haplotypes we define a vector with elements *h*_*i*_ with 1 ≤ *i* ≤ *p* whose *i*th element is the frequency of the *i*th haplotype. The vector **h** is then used to define another vector **c** = **h** ^*t*^ **A**. The number of elements of **c** is equal to the number of animals. Values of **c** are larger for animals which carry many more frequent haplotypes. Thus the values of **c** will be used as a guide to select animals when sequencing resources are limited. In addition, it is important to ensure that the same haplotype is not sequenced in a large number of different animals so as to maximize the haplotype diversity in the animals selected for sequencing. All these different requirements can be summarized in the following set of inequalities.

$$\begin{array}{ccc} \text{maximize}\; & z_{2}=\overset{n}{\underset{j=1}{\sum }}c_{j}x_{j},\;x_{j}\in 0,1 & \end{array}$$ (5)

$$\begin{array}{ccc} \text{subject to}\; & \overset{n}{\underset{j=1}{\sum }}x_{j}\leq n_{max} & \end{array}$$ (6)

$$\begin{array}{ccc} & \overset{n}{\underset{j=1}{\sum }}a_{ij}x_{j}\leq r_{max}\;i=1,2,3,\cdots \;,p & \end{array}$$ (7)

Equation 5 is the objective function to maximize the number of more frequent haplotypes represented by the selected animals. Equation 6 is the constraint to set the maximum number of animals to be sequenced to be equal to *n*_*max*_. Equation 7 is the constraint to ensure that each haplotype covered is at the most covered by *r*_*max*_ animals, where *r*_*max*_ should be a positive integer ≥ 1 and is chosen to be considerably smaller than *n*_*max*_. Ideally, the objective function should be maximized with *r*_*max*_ chosen to be small and the maximization performed in one step. This would permit the optimal selection of multiple animals without introducing any approximations. In our data analysis, for reasons which will soon be discussed, we will introduce a number of additional approximations in the solution to Eqs 5–7.

The form of Eqs 5–7 are generic in a linear programming context, and in a broader context the coefficients *c*_*j*_ in Eq. 5 may be freely chosen, as long as the coefficients are positive. With the coefficients chosen as described in Butty et al. (2019), it is possible to prioritize the selection of animals in the IWS scheme (Bickhart et al., 2016). The highly segregating haplotype (HSH) scheme mentioned in Butty et al. (2019) uses coefficients similar to the ones we suggest, but with some additional multiplicative factors which are introduced to avoid including the same haplotype in multiple animals. In our framework, this is achieved by the choice of *r*_*max*_. One important issue addressed in Bickhart et al. (2016), how to select the smallest number of samples needed to sequence all haplotypes above a given frequency threshold, can be addressed within the framework of linear programming by solving Eqs 3, 4 after discarding the rows corresponding to the low frequency haplotypes. If no rows, or very few rows are discarded in Eqs 3, 4, then animals containing rare haplotypes can be targeted, which is one of the objectives of the optimized GDI method discussed in Butty et al. (2019). Thus many of the main key ideas for the prioritization of animals in Bickhart et al. (2016) and Butty et al. (2019) can be incorporated into a Linear Programming framework.

Linear programming problems described above cannot be solved analytically, however branch and bound methods (Land and Doig, 1960) are guaranteed to converge to the global optimum. In practice, for reasons which will be discussed later, the convergence can be very slow without the use of approximations. It turns out that for the first application Eqs 1, 2 can be solved rapidly and exactly. No approximations are needed and the identification of which animals to sequence with a view to include all haplotypes is done in LPChoose in a single step. In Gonen et al. (2017) the authors also comment on the possibility of determining which animals are needed to cover all haplotypes, but through the solution of a system of equations in multiple stages.

In the case of the second application, it turns out that convergence in LPChoose is very slow if exact solutions are sought. In order to facilitate convergence in the second application we will set the value of *r*_*max*_ to 2 and instead of selecting all *n*_*max*_ animals at once, we will first select 2 animals (i.e., *n*_*max*_ = 2). Once the first two animals have been identified, these animals and all the haplotypes present in them are removed. Then the optimization in Eqs 5–7 is repeated but with the objective function in Eq. 5 suitably modified to reflect the absence of the first two animals and also the haplotypes that they carry. This procedure can be carried out until the desired number of animals has been found. This approach amounts to breaking up the larger optimization, which cannot be solved exactly in reasonable time, into a smaller number of problems each of which can be solved exactly and quickly by linear programming, and then combining the results.

In Bickhart et al. (2016), both the AHAP selection schemes, AHAP1 and AHAP2, use the same weights as LPChoose, those in Eq. 5, but consider only homozygous haplotypes. Furthermore, in the AHAP1 scheme, there is no updating of weights to take into account animals already selected. We will present results with no updating of weights, as in AHAP1, but using both homozygotes and heterozygotes. Our results for AHAP1 will use *n*_*max*_ = 1 repeatedly until the desired number of animals is obtained. Even though the AHAP2 scheme in Bickhart et al. (2016) uses the same weights and updating as LPChoose, there is an important difference between LPChoose and AHAP2 as implemented in Bickhart et al. (2016). LPChoose is able to select multiple animals at a time in contrast to the AHAP2 scheme in Bickhart et al. (2016).

In the results we present for IWS, we will prioritize the animals using the IWS weighting scheme in Bickhart et al. (2016) and Butty et al. (2019), and select a single animal at a time, in order to facilitate a comparison with the IWS implementation in Bickhart et al. (2016). Multiple rounds of selection are performed until the desired number of animals is obtained.

In order to compare the different methods described, genotype data for five different scenarios were simulated following the general scheme for simulating artificial populations in Gonen et al. (2017). The number of generations considered in these 5 scenarios are 5, 10, 15, 30, or 50. These scenarios resemble modern cattle populations. The genome of 10,000 segregating loci on 10 chromosomes was simulated using the “cattle genome” option in AlphaSimR (Gaynor et al., 2020). A quantitative trait controlled by 150 QTL of effects sampled from standard normal distributions distributed equally on 10 chromosomes was simulated. First, founders of 1,000 cattle of equal sex ratio were generated. At each generation, the best 25 males were selected as sires on the basis of their highest breeding value and mated to all 500 females as dams to produce next generations with 1,000 cattle of equal sex ratio. In our analysis, 10 replicates were simulated for each of the five scenarios. From these simulations, all individuals had haplotypes for 10,000 SNPs distributed equally across the 10 chromosomes. In each population, haplotype blocks of length 100 SNPs were obtained across 10 chromosomes. A mismatch of up to 10% was used to ensure that haplotypes with small differences were considered as identical.

In the results, the selection of a fixed number animals will also be made after the exclusion of haplotypes with a frequency of less than 1%, similar to the strategy adopted in Su et al. (2014). For the rest of the manuscript, haplotypes which are retained after this exclusion will be referred to as common haplotypes. The results in Bickhart et al. (2016) are also based on the exclusion of low frequency haplotypes but with a more drastic restriction on haplotype frequency than in Su et al. (2014). A brief description of the simulation scenarios is presented in Table 1. We will also briefly discuss results in which the number of animals in Table 1 remains the same but the number of haplotypes is considerably increased. An open-source, publicly available Julia package called LPChoose (https://github.com/reworkhow/LPChoose.jl) which makes use of the GNU Linear Programming Kit (GLPK) has been developed. For the sake of the reproducibility of our results, we make no use whatsoever of any proprietary solvers for linear programming.

**TABLE 1:** Simulation scenarios.

Scenario	Total number of animals	Number of unique haplotypes	Number of unique common haplotypes
1	6,000	27,399 ± 332	2,638 ± 42
2	11,000	37,922 ± 758	2,376 ± 50
3	16,000	44,591 ± 826	2,216 ± 49
4	31,000	56,036 ± 1,313	1,959 ± 54
5	51,000	61,418 ± 2,023	1,815 ± 44

## 3 Results

The minimum numbers of animals identified in LPChoose, AlphaSeqOpt, IWS, and AHAP to cover all unique haploptypes in five populations are shown in Table 2. The results for AlphaSeqOpt in the third column of Table 2 were obtained from repeated runs of AlphaSeqOpt with varying numbers of focal animals as defined in Gonen et al. (2017) to be selected. In the case of IWS and AHAP, animals were selected one at a time until all haplotypes were covered. For these methods, the selection of successive animals was done using the weights discussed earlier. The minimum numbers of animals identified by LPChoose were consistently smaller than those from AlphaSeqOpt, IWS, and AHAP. The results of Table 2 suggest that the difference between LPChoose and the other methods in the smallest number of animals increases as the size of the population increases.

**TABLE 2:** Minimum number of animals identified representing all haplotypes in the population.

Scenario	Minimum number of animals (mean ± SD)			
	LPChoose	AlphaSeqOpt	IWS	AHAP
1	4,086 ± 13	4,988 ± 30	4,154 ± 30	5,998 ± 2
2	6,663 ± 18	8,563 ± 51	6,802 ± 84	10,999 ± 2
3	8,648 ± 34	11,563 ± 132	8,821 ± 75	15,998 ± 3
4	12,229 ± 86	17,506 ± 275	12,738 ± 225	30,997 ± 6
5	14,241 ± 119	21,098 ± 494	15,028 ± 356	50,999 ± 1

We emphasize that the results in column 2 of Table 2 obtained from LPChoose are obtained without any approximations, and are thus equal to the theoretically lowest values attainable for this kind of problem. Furthermore, when LPChoose is used to select the minimum number of animals to cover all haplotypes, some haplotypes may be covered by multiple animals. This introduces a certain level of redundancy in sequencing which may be desirable when certain haplotypes should be covered more frequently for facilitating imputation (Ros-Freixedes et al., 2017).

LPChoose, AlphaSeqOpt, IWS, and AHAP were also used to select a fixed number of animals (100 animals) whose haplotypes represent the maximum proportions of the total haplotypes in the population. The results obtained are shown in Tables 3, 4. In Table 3, the proportion of all haplotypes (with no lower limit on haplotype frequencies) represented by 100 selected animals were compared. In this scenario IWS performed best in terms of haplotype coverage, followed by LPChoose. However, independent of the method only a small proportion (0.1–0.3) of all haplotypes were covered.

**TABLE 3:** Proportion of all haplotypes represented by 100 selected animals in the population.

Scenario	Proportion of all haplotypes (mean ± SD)			
	LPChoose	AlphaSeqOpt	IWS	AHAP
1	0.2218 ± 0.0036	0.2052 ± 0.0048	0.2504 ± 0.0032	0.1441 ± 0.0041
2	0.1700 ± 0.0020	0.1528 ± 0.0048	0.1951 ± 0.0024	0.1007 ± 0.0024
3	0.1498 ± 0.0026	0.1319 ± 0.0047	0.1735 ± 0.0009	0.0805 ± 0.0029
4	0.1249 ± 0.0032	0.1119 ± 0.0037	0.1464 ± 0.0022	0.0584 ± 0.0024
5	0.1145 ± 0.0027	0.1084 ± 0.0036	0.1370 ± 0.0023	0.0435 ± 0.0025

**TABLE 4:** Proportion of common haplotypes represented by 100 selected animals in the population.

Scenario	Proportion of common haplotypes (mean ± SD)			
	LPChoose	AlphaSeqOpt	IWS	AHAP
1	0.9934 ± 0.0017	0.9239 ± 0.0032	0.9917 ± 0.0017	0.8520 ± 0.0159
2	0.9911 ± 0.0028	0.9041 ± 0.0071	0.9899 ± 0.0017	0.8288 ± 0.0142
3	0.9921 ± 0.0018	0.8766 ± 0.0171	0.9875 ± 0.0032	0.7625 ± 0.0257
4	0.9899 ± 0.0021	0.8444 ± 0.0109	0.9780 ± 0.0030	0.7004 ± 0.0247
5	0.9993 ± 0.0006	0.8335 ± 0.0088	0.9600 ± 0.0061	0.6287 ± 0.0336

In Table 4, the proportion of common haplotypes represented by 100 selected animals were compared. This scenario is similar to that discussed in Bickhart et al. (2016). LPChoose performed better than the other three methods consistently across different populations, and the proportions of common haplotypes identified in LPChoose were usually higher than 0.99, followed by IWS with slightly lower proportions.

The better results obtained by using the IWS weighting scheme in Table 3 are to be expected since IWS preferentially selects animals with low-frequency haplotypes by assigning larger weights to individuals containing rare haplotypes (Bickhart et al., 2016), unlike LPChoose where more common haplotypes are preferred. The prioritization of animals in IWS, however, can be easily accommodated in a Linear programming framework by changing the weights in Eq. 5.

All the results discussed so far were obtained in 10 min or less of execution time. If the number of haplotypes is increased five fold compared with our original simulations keeping the number of animals unchanged, there is a five fold increase in running times for LPChoose in application 1 and a ten fold increase for application 2. Our earlier conclusions about the relative merits of the different methods considered remain unchanged; for the analyses in Table 2 and in Table 4, LPChoose still performs best.

## 4 Discussion

In this paper, linear programming methods are used to answer two questions for the allocation of sequencing resources, to identify the minimum number of animals whose haplotypes represent all the haplotypes in the population, and to choose for sequencing a fixed number of animals whose haplotypes represent the maximum proportion of the common haplotypes in the population. The results from Tables 2, 4 suggest that linear programming methods, which permit the selection of more than one animal at a time, may still offer a number of advantages in comparison with other methods which rely on the selection of a single animal at a time. It is noteworthy that these improvements were obtained using relatively straightforward approximations, and a publicly available implementation of linear programming.

We make no explicit use of pedigree information, which may be important in deciding which animals to sequence (Boichard, 2002; Ros-Freixedes et al., 2020). If the animals to be sequenced should also be selected based on positions in the pedigree, then additional requirements based on pedigree structure can be incorporated in a Linear Programming framework.

The expected accuracy of genotype imputation has also been used as a criterion to determine the best animals to be sequenced (Ros-Freixedes et al., 2020; Yu et al., 2014). Genotype imputation accuracy is expected to improve when a larger number of animals are sequenced at lower coverage as opposed to a smaller number of animals at high coverage (keeping the product of read depth and number of sequenced animals fixed) (VanRaden et al., 2015). Our results suggest that for a fixed number of animals, LPChoose covers more haplotypes than competing methods, suggesting that selecting animals through Linear Programming could permit a higher level of genotype imputation accuracy.

In Bickhart et al. (2016), the authors point out that the accuracy of imputation of rare variants is affected by the lower limit put on haplotype frequencies and suggest the importance of including additional animals to improve the accuracy of imputation of rare variants. In Bhati et al. (2020) as well, the authors comment on how the inclusion of rare variants is affected by the choice of animals sequenced. The linear programming methods we present here can be adapted to the selection of multiple animals with low frequency haplotypes by modifying the right hand sides of Eqs 6, 7 to increase the values of *r*_*max*_ in Eq. 7 for those rows corresponding to rare haplotypes. The selection of multiple animals with low frequency haplotypes can also be achieved in the framework of Method 1, by increasing the right hand side of Eq. 2 for those rows corresponding to low frequency haplotypes.

Furthermore, if additional animals with previously known sequence information are to be included in the set of animals to be sequenced, these animals can be included through additional constraints in Eq. 4. Thereafter, any additional selection can be carried out while maximizing the information present in the animals included by requirement.

There are some unavoidable limitations associated with the linear programming methods which necessitate the use of approximations discussed earlier. To understand the necessity of approximations it is useful to use graph theory to rephrase the first problem we address as that of finding a minimum set cover on a bipartite graph *via* integer linear programming (Vazirani, 2003). The problem of finding a suitable subset of animals with common haplotypes, can also be rephrased as a set cover problem along with additional weighting factors. As there is no known polynomial time algorithm for solving the set cover problem approximations are unavoidable in large data sets. Greedy approximations which select a single element (or animal in the context of sequencing) at a time are relatively straightforward to implement, but frequently do not lead to the true optimum (Vazirani, 2003). Hence methods which rely on the selection of more than one animal at a time, such as those we have presented here, may lead to improved results.

To conclude, we have illustrated the use of linear programming for optimizing the allocation of sequencing resources. Our results suggest how linear programming can be used to extend and improve the approximations used in Butty et al. (2019) and Bickhart et al. (2016) to address the important questions discussed in these papers. It is encouraging that the superior results found by LPChoose in Tables 2, 4 were obtained without using proprietary linear programming solvers, and applying straightforward heuristics when necessary. The use of more sophisticated heuristics in conjunction with proprietary solvers could lead to additional improvements in both efficiency and lowered running time.

## Data Availability

The original contributions presented in the study are included in the article/Supplementary Material, further inquiries can be directed to the corresponding author.
